# Global Changes in DNA Methylation in Seeds and Seedlings of *Pyrus communis* after Seed Desiccation and Storage

**DOI:** 10.1371/journal.pone.0070693

**Published:** 2013-08-05

**Authors:** Marcin Michalak, Mirosława Z. Barciszewska, Jan Barciszewski, Beata P. Plitta, Paweł Chmielarz

**Affiliations:** 1 Polish Academy of Sciences, Institute of Dendrology, Kórnik, Poland; 2 Polish Academy of Sciences, Institute of Bioorganic Chemistry, Poznań, Poland; University of Leeds, United Kingdom

## Abstract

The effects of storage and deep desiccation on structural changes of DNA in orthodox seeds are poorly characterized. In this study we analyzed the 5-methylcytosine (m^5^C) global content of DNA isolated from seeds of common pear (*Pyrus communis* L.) that had been subjected to extreme desiccation, and the seedlings derived from these seeds. Germination and seedling emergence tests were applied to determine seed viability after their desiccation. In parallel, analysis of the global content of m^5^C in dried seeds and DNA of seedlings obtained from such seeds was performed with a 2D TLC method. Desiccation of fresh seeds to 5.3% moisture content (mc) resulted in a slight reduction of DNA methylation, whereas severe desiccation down to 2–3% mc increased DNA methylation. Strong desiccation of seeds resulted in the subsequent generation of seedlings of shorter height. A 1-year period of seed storage induced a significant increase in the level of DNA methylation in seeds. It is possible that alterations in the m^5^C content of DNA in strongly desiccated pear seeds reflect a reaction of desiccation-tolerant (orthodox) seeds to severe desiccation. Epigenetic changes were observed not only in severely desiccated seeds but also in 3-month old seedlings obtained from these seeds. With regard to seed storage practices, epigenetic assessment could be used by gene banks for early detection of structural changes in the DNA of stored seeds.

## Introduction

Plants modulate their physiology and development through genome-wide changes in gene expression in response to environmental conditions [1]. The mechanisms of desiccation tolerance of orthodox (desiccation tolerant) seeds are well known. These mechanisms include the accumulation of late embryogenesis abundant proteins (LEA), dehydrins [2], non-reducing sugars, sucrose [3], selenium [4], activation of antioxidants [5], [6], intracellular dedifferentiation [7], metabolic ‘switching off’ [8], and the operation of repair systems [9], [3]. Although a number of phenomena, as discussed above, have been implicated in the acquisition and maintenance of desiccation tolerance, a coherent view integrating control of the acquisition of desiccation tolerance still remains to be elucidated [10].

It has been recently shown that epigenetic regulation of gene expression is involved in plants’ response to environmental stresses (i.e. drought stress) [1]. The most extensively studied and characterized epigenetic modification of DNA is the methylation of cytosine, which represents the addition of a methyl group to carbon 5 (C5) of the pyrimidine ring [11]. DNA methylation in plants predominantly occurs at CG dinucleotides, but also occurs at CHG (where H is A, C or T) and asymmetric CHH (where H is A, C or T) sites [12].

At the present time, the relationship of this epigenetic modification to the adaptation of seeds to environmental stress is not entirely understood. Nevertheless, it is apparent that plants modulate their physiology and development through epigenetic modifications of their DNA [1]. Specifically, DNA methylation and histone modification, play a crucial role in the regulation of gene expression during the responses of mature plants to environmental stresses [13], [14], [15]. Furthermore, these modifications can be inherited by subsequent generations [16], [17]. In plants, the methylation of symmetric (CG, CHG) and asymmetric (CHH) sequences is maintained by DNA methyltransferases: Methyltransferase 1 (MET 1) [18], [19], [20], Chromomethylase3 (CMT3) [21] and DNMT3a/3b homologs: Domains Rearranged Methylase 1 and 2 (DRM1/2), which require an active guiding by small interfering RNAs (siRNAs) to target DNA [22], [23].

Orthodox seeds are dried to specific moisture content before they are stored in gene banks that have been established to preserve the seeds under conditions that ensure genetic stability [24]. We hypothesize that seed moisture content and temperature affect the course of epigenetic changes, including the global methylation of genomic DNA. Understanding the stresses that affect the overall level of DNA methylation in seeds and during seedling development is crucial for effective storage of genetic resources in *ex situ* gene banks.

Most of the stress-induced alterations in epigenetic modification of DNA are reset upon relief from the stress. However, in non-Mendelian inheritance, some of the modifications are stable and may be carried forward in the form of a ‘stress memory’ that is inherited across mitotic or even meiotic cell divisions. It is thought that epigenetic stress memory may help plants to cope more effectively with subsequent stresses [25]. However, the effects of stresses on the level of DNA methylation in seeds have not been previously analyzed, and it is not known whether epigenetic changes are induced. We hypothesized that DNA methylation levels are altered at the whole-genome level, as well as at the level of individual genes, in response to environmental stresses. It is likely that alterations in the global level of DNA methylation could play an important role in the response to desiccation of orthodox seeds. It may be relevant because they typically undergo deep desiccation in gene banks to inhibit their aging and prolong storage.

In the present study, we analyzed the effects of desiccation and storage on the global level of DNA methylation of common pear (*Pyrus communis* L.) seeds. In addition, the abundance of m^5^C in seedlings obtained from these seeds was also analyzed. Influence of desiccation on orthodox seed germination, seedling emergence and growth was assessed as well.

## Materials and Methods

### Ethics Statement

No specific permits were required for the described experimental field studies: a) no specific permissions were required for these locations b) locations are not privately-owned or protected; c) the field studies did not involve endangered or protected species.

### Plant Material

Seeds of common pear (*Pyrus communis* L.*)* were collected when ripe after abscission (green turning to yellow) in 2007 from Łopuchówko (52.6151°N, 17.0896°E; 30 km north of Poznań) and in 2009 from Borkowice (52.2167°N, 16.8000°E; 30 km south of Poznań), Poland. Seeds were extracted manually from fruits. Collections from Borkowice were represented by fresh seeds (non-stored) and seeds stored for 1 year. For collections from Łopuchówko, only seeds stored for 2 years were used in the experiment. For both provenances, partially dried seeds were stored at 3°C.

### Seed Moisture Content

After harvest, seeds were dried on a laboratory bench at 20°C to a moisture content (mc) of 8.2–8.5% (safe mc, used later as a control for severely desiccated seeds of mc 2.2–2.8% from both provenances). Desiccation to the lowest levels of seed mc, i.e. 2.2% and 2.8%, was performed over activated silica gel in a tightly closed vessel. The required seed mass, corresponding to the measured mc, was calculated using the following formula [26]:
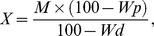
where *X* = final mass of the seeds

*M* = fresh mass of the seeds

*Wp* = moisture content of seeds before drying

*Wd* = desired moisture content of seeds.

Seeds were placed in a drying box on blotting paper (in a layer with a thickness not exceeding two times the seed height) and desiccated for 14 d. The mc of seeds was assessed with three replications of 10 seeds each by drying at 103°C±2°C for 18 h. Moisture content was expressed as a percentage on a fresh-weight basis. Stratification of the dormant seeds was required before subsequent germination and seedling emergence tests.

### Stratification

The seeds were placed in plastic boxes in a moist substrate mixture (1∶1, v/v) of quartz sand (<1 mm fraction) and sieved peat with pH 3.5–4.5 [27]. Seeds were mixed with the substrate (1∶3, v/v) and placed in 0.25 L plastic bottles. Seeds were monitored for fungal infections and the first germinated seeds were counted. Specifically, seeds were visually assessed for the emergence of a radicle (2–3 mm long), which is a visible indicator of release from dormancy. The substrate was watered weekly if required and seeds were stratified for 16 weeks at 3°C, until no more than 5% of seeds had developed a visible radicle. Subsequently, the seeds were transferred to a cyclically alternating temperature regime and subjected to a germination test [26].

### Germination, Seedling Emergence Test, and Seedling Growth

Stratified seeds were germinated in darkness, in an identical substrate mixture that used for stratification. Optimum thermal conditions for seed germination were ensured by cyclically alternating temperatures of 3°C/20°C (16 h/8 h per day, respectively). Under these conditions, a seed with a 3 mm long radicle was considered as “germinated”.

After the germination test, a final evaluation of seed viability was performed and seeds were dissected for visually assessment. The seeds were cut with a scalpel along the longitudinal axis, across the cotyledons and the embryonic axis. All seeds that failed to germinate by the end of the testing period were classified as “decayed seeds”.

Seedling emergence was also evaluated with an identical substrate that was used for stratification and the germination tests. Stratified seeds were sown in plastic boxes in the substrate at a depth of 1 cm, and covered with a layer of sand. The boxes were covered with a transparent lid to enable the penetration of light to emerging seedlings and to maintain a suitable moisture level. The lid was subsequently removed when seedlings were approximately 2–3 cm high. Thermal conditions were identical to the germination test (3°C/20°C, 16 h/8 h per day) until the emergent seedlings were approximately 2–3 cm high. Boxes with seedlings were then moved into a controlled growth chamber at 25°C under light conditions (16 h/8 h photoperiod at 60 µmolm^−2^s^−1^) and maintained for 4–5 weeks.

For epigenetic analysis (determination of the m^5^C content in leaf DNA), seedlings were grown for 3 months under a 16 h/8 h photoperiod at 77 µmol m^−2^ s^−1^at 20°C. Each experiment was comprised of four replicates, containing 50 seeds each.

The height of 3-month-old seedlings was measured for seeds that were harvested from the Borkowice provenance. The seeds were stored for 1 year and had an 8.5% mc. After storage, they were desiccated to 2.2% mc and used for subsequent seedling growth assays. Shoot height (mm) was measured from the root collar to the shoot apex of germinated seedlings. Each experiment was replicated 4 times and contained 25 seedlings per replicate experiment.

### DNA Isolation and Assessment of Global DNA Methylation Levels

Total genomic DNA was extracted from embryos (after removal of the seed coat) and leaves of seedlings with the Qiagen DNAeasy Plant Mini Kit (Qiagen, Hilden, Germany). One variant of the experiment (DNA extraction) consisted of 3 replicates. Each replicate consisted of five embryos. For DNA extraction from leaves, for one replicate one-quarter of an apical part of three leaves from one seedling were used. The level of m^5^C was measured five times for each biological replicate.

Analysis of the global content of m^5^C in DNA was carried out as previously described [28]. This method has been already successfully applied for the assessment of DNA methylation in plants [29]. Although it is currently possible to assess global methylation status with HPLC analyses, there are some serious technical limitations to this approach. HPLC requires a substantial amount of the starting material and DNA preparations must be RNA-free since it is a technical challenge to use column chromatography to differentiate the ribo- and deoxyribonucleoside of 5-methylcytosine. This consideration is especially challenging in case of DNA samples that are derived from seeds that are rich in storage material. A thin-layer chromatography (TLC) based method seems to be the most appropriate since it requires a small amount of DNA and ribo- and deoxyribonucleotides are easily distinguishable under this experimental testing condition. Moreover, even small changes in 5-methylcytosine amount are capable of being detected [28].

To begin our evaluation of global DNA methylation level, we first dried DNA (1 µg) and then digested with 0.001 U spleen II nuclease and 0.02 U micrococcal nuclease in 20 mM succinate buffer containing 10 mM CaCl_2_ for 6 h at 37°C. The hydrolysate (0.3 µg) was then labelled with 1.6 µCi [γ-^32^p]ATP (6,000 Ci/mmol; Hartmann Analytic, Germany) and 1.5 U T4 polynucleotide kinase in 10 mM bicine-NaOH buffer (pH 9.7) containing 10 mM MgCl_2_, 10 mM dithiothreitol, and 1 mM spermidine. After incubation for 30 min at 37°C, 0.03 U apyrase in 10 mM bicine-NaOH buffer was added and incubated for 30 min. Next, 0.2 µg RNase P1 in 500 mM ammonium acetate buffer (pH 4.5) was used for 3′-phosphate cleavage. The [γ-^32^p] m^5^C content was analyzed by two-dimensional thin layer chromatography on cellulose plates (Merck) in isobutyric acid: NH_4_OH:H_2_O (66∶1∶17) and 0.1 M sodium phosphate (pH 6.8)–ammonium sulfate–*n*-propanol (100 mL/60 g/1.5 mL, v/w/v). Radioactivity was subsequently measured using a Fluoro Image Analyzer FLA-5100 with Multi Gauge 3.0 software (FujiFilm). Each analysis was repeated for a total of four times.

**Figure 1:**
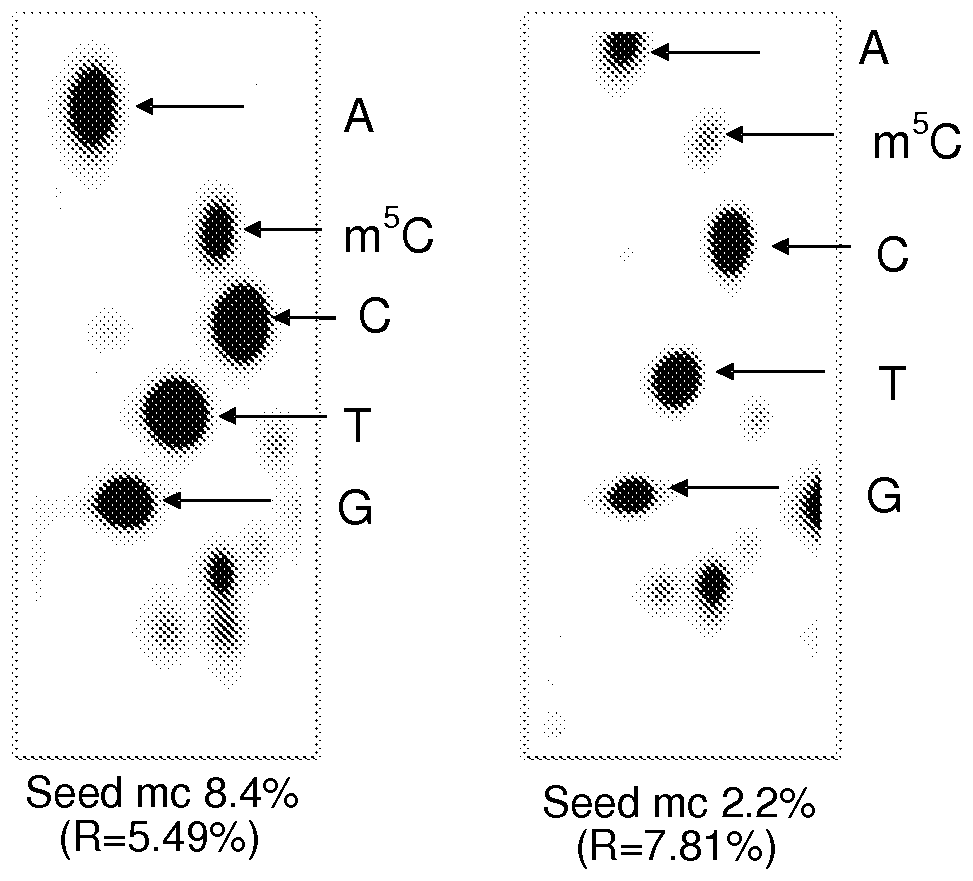
Assessment of global DNA methylation of seeds from Borkowice, stored for 1 year. 2′deoxynucleotides derived from DNA hydrolysis (labelled spots) and RNA contamination (unlabelled spots) are clearly separated. A- adenine, m^5^C -5-methylcytosine, C-cytosine, T-thymine, G- guanine.

For quantitative evaluation of m^5^C content, fluoroscopic image analyses accounted for the contents of C, T and m^5^C. The global level of m^5^C (R) was calculated with the following formula:
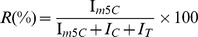
where *I* is the intensity of individual spots corresponding to the nucleotide analyzed (Figure1).

### Statistical Analysis

STATISTICA software, 1998 edition (StatSoft Polska, 1995–2005) was used for the statistical analyses of all data. Analysis of variance (ANOVA) was used to determine the significance of differences between means, and a Tukey’s test was used for pair-wise comparisons. The Tukey’s test was performed after arc-sine transformation and was always run at a significance level of *P*≤0.05. Separate ANOVAs and Tukey’s tests were performed for analysis of germination, seedling emergence, global methylation, and seedling height. Standard deviations were indicated as errors bars on graphs.

## Results

**Figure 2:**
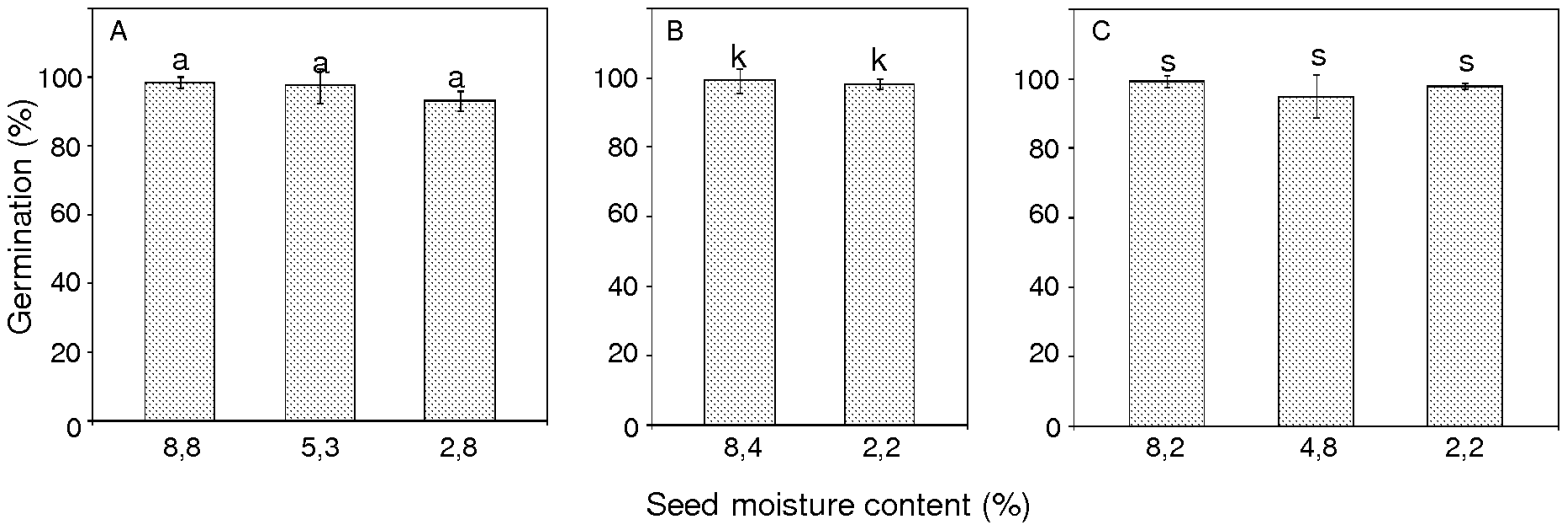
Effects of seed desiccation and storage on the germination of pear seeds. (A) Unstored seeds from Borkowice. (B) Seeds from Borkowice that had been stored for 1 year. (C) Seeds from Łopuchówko that had been stored for 2 years. Values marked with the same letter are not significantly different at *P*<0.05, ANOVA, Tukey`s test, error bars indicate standard deviations.

The germination of control seeds, which were not deeply desiccated and had a moisture content of 8.2–8.8%, was nearly 100% (Figure 2). Additional desiccation of freshly collected seeds over silica gel to a 5.3% mc (Borkowice) or 4.8% mc (Łopuchówko) did not significantly affect seed germinability. Seeds from both provenances that were desiccated to 2.2–2.8% mc showed a 90–98% germination rate (Figure 2).

**Figure 3:**
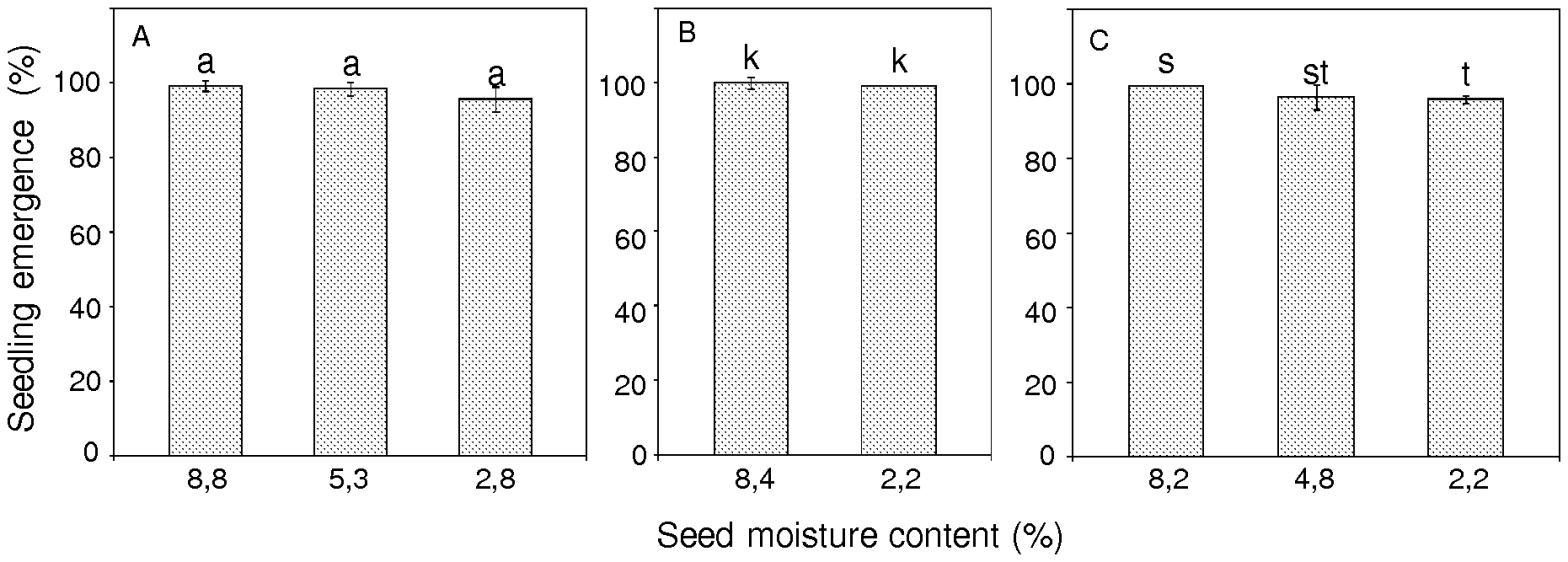
Effects of pear seed desiccation and storage on seedling emergence. (A) Unstored seeds from Borkowice. (B) Seeds from Borkowice that had been stored for 1 year. (C) Seeds from Łopuchówko that had been stored for 2 years. Values marked with the same letter are not significantly different at *P*<0.05, ANOVA, Tukey`s test, error bars indicate standard deviations.

Control seeds from the two provenances with 8.2, 8.4, or 8.8% mc showed 100% seedling emergence (Figure 3). Emergence was categorized as emergent plants containing several leaves and an average height approximately 3 cm. Desiccation of seeds from the two provenances to 4.8% (2-years stored seeds) or 5.3% mc (fresh seeds) did not significantly reduce the level of seedling emergence, which remained high at 98–100% (Figure 3). Similarly, desiccation of 1-year stored seeds from Borkowice to 2.2 or 2.8% mc did not induce significant changes and a similar percentage of seedling emergence was observed (98–100%). However, desiccation of seeds from Łopuchówko to 2.2% mc resulted in a significant reduction of seedling emergence from 100 to 96% in comparison to control seeds (8.2% mc) (Figure 3). For all sets of seeds that were studied, all were considered equal in both quality and viability.

### Methylation of DNA after Seed Desiccation

**Figure 4:**
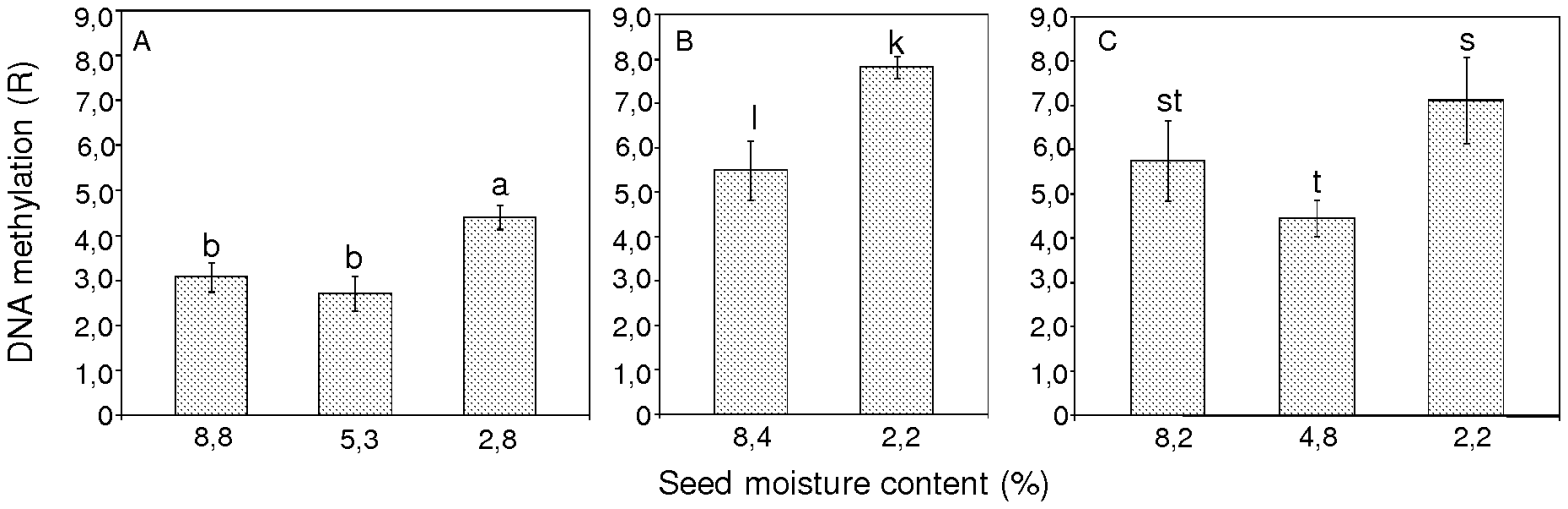
Changes in the percentage of DNA in pear seeds that was modified to carry 5-methylcytosine after desiccation and storage. (A) Unstored seeds from Borkowice. (B) Seeds from Borkowice that had been stored for 1 year. (C) Seeds from Łopuchówko that had been stored for 2 years. Values marked with the same letter are not significantly different at *P*<0.05, ANOVA, Tukey`s test, error bars indicate standard deviations.

The global level of DNA methylation (R-percentage of methylated cytosines) was R = 3.08% for seeds that were desiccated to 8.8% mc immediately after harvest. Further drying of the seeds to 5.3% mc resulted in a slight reduction in the level of methylation to R = 2.73% (Figure 4A). Deep drying of seeds over silica gel to 2.8% mc significantly increased the level of m^5^C to R = 4.42% in comparison to seeds with 5.3 or 8.8% mc (Figure 4A).

Control seeds (8.4% mc) that were stored for 1 year prior to deep desiccation contained a high level of m^5^C (R = 5.49%) (Figure 4B), which was higher than that of DNA in fresh seeds (8.8% mc; R = 3.08%), (Figure 4A). The drying of seeds after 1-year storage to 2.2% mc resulted in an increase in the global DNA methylation level from R = 5.49 to 7.81% in comparison to seeds that were not as deeply desiccated, 8.4% mc (Figure 4B).

Seeds from the Łopuchówko provenance (stored for 2 years at 8.2% mc) were characterized by R = 5.75% immediately after storage (Figure 4C). Drying of the seeds to 4.8% mc slightly reduced the m^5^C content from R = 5.75 to 4.45%, whereas further drying to 2.2% mc resulted in a significant increase in m^5^C content to R = 7.11% (Figure 4C). For stored seeds, the global level of m^5^C (R = 4.5–7.5%) (Figure 4B, C) was higher than that of non-stored seeds (R = 2.8–4.5%), (Figure 4A).

**Figure 5:**
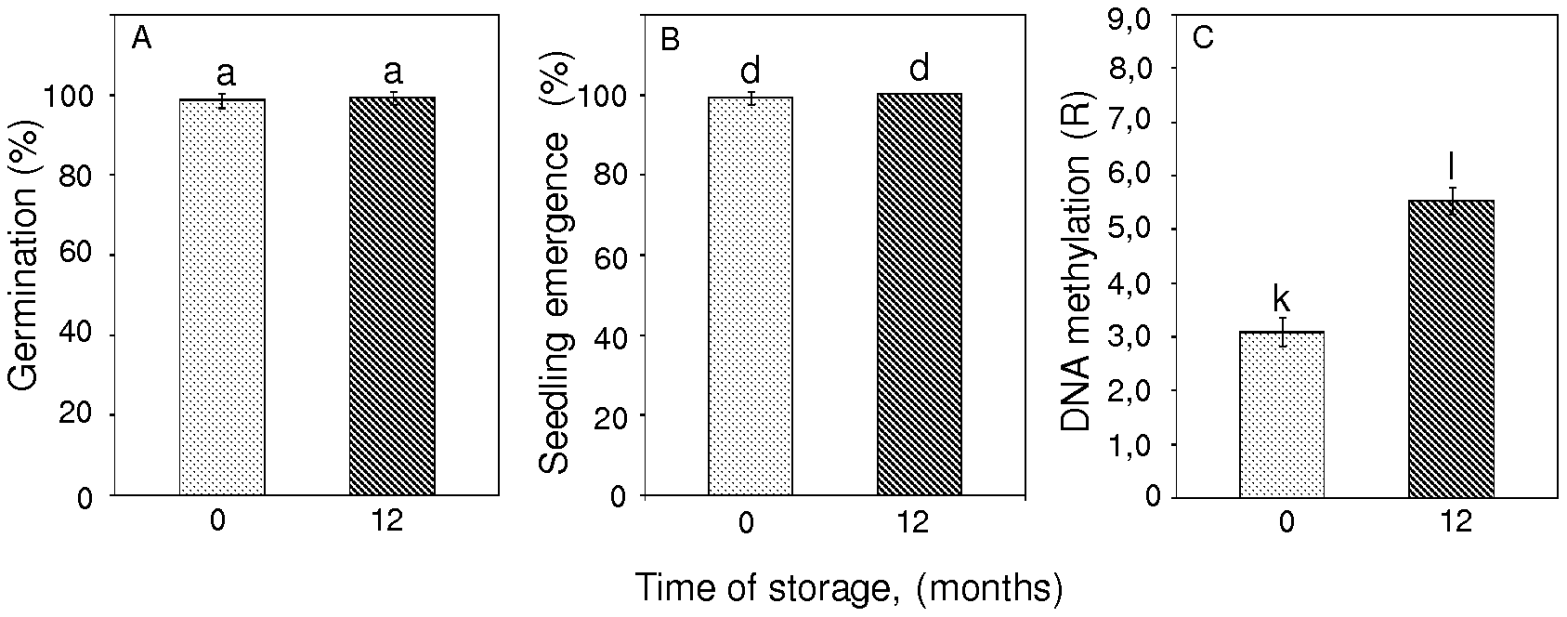
Changes in germination (A), seedlings emergence (B) and DNA methylation level (C) in seeds from Borkowice (8.8% mc) after 12 months of storage. Values marked with the same letter are not significantly different at *P*<0.05, ANOVA, Tukey`s test, error bars indicate standard deviations.

No significant differences were observed for the germination and emergence of seeds that were analyzed freshly after harvest from Borkowice (8.8% mc) or after a 1 year duration of storage (8.4% mc), (Figure 5). However, a comparison of the percentage of DNA methylation revealed a relevant time-dependent increase in m^5^C. After 1 year in storage, the amount of 5-methylcytosine increased from 3.08 to 5.49% (Figure 5).

### Seedlings Growth

**Figure 6:**
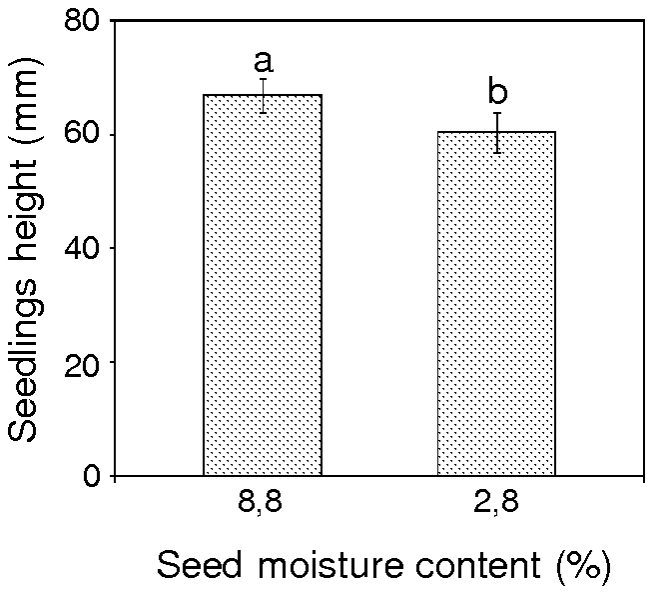
Height of seedlings (mm) derived from desiccated pear seeds. Values marked with the same letter are not significantly different at *P*<0.05, ANOVA, Tukey`s test. Seeds collected from Borkowice were stored for 1 year at 3°C before desiccation, error bars indicate standard deviations.

Three-month-old seedlings that were derived from seeds dried to 2.8% mc were significantly shorter (mean height 61.5 mm) than similar aged seedlings that were generated from control seeds (8.8% mc; mean height 66.6 mm), (Figure 6).

### DNA Methylation in Seedlings Derived from Desiccated Seeds

**Figure 7:**
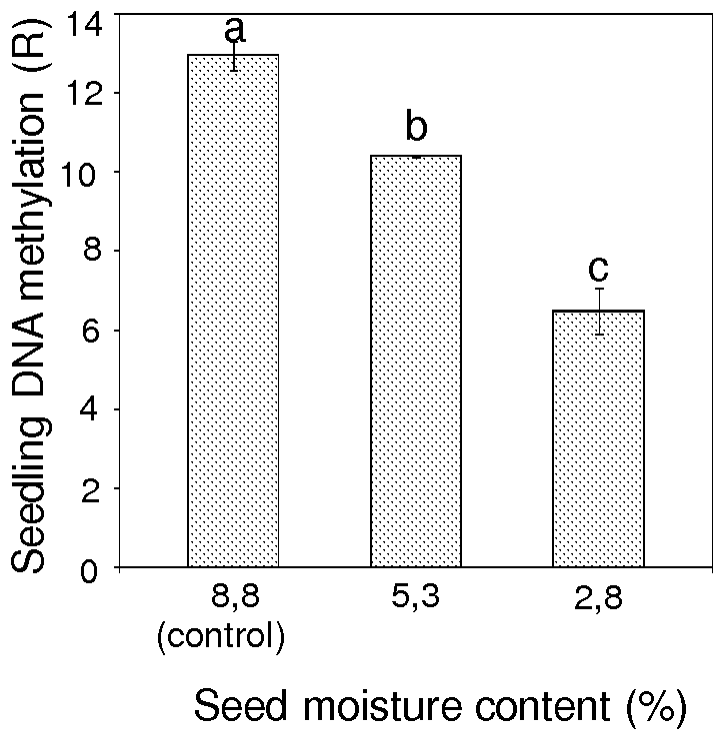
Percentage 5-methylcytosine methylation of DNA in seedlings derived from desiccated pear seeds. Values marked with the same letter are not significantly different at *P*<0.05, ANOVA, Tukey`s test. Seeds were collected from Borkowice and not stored before desiccation, error bars indicate standard deviations.

The global DNA methylation level in seedlings obtained from seeds desiccated to different levels of mc (2.8–8.8%) ranged from R = 6.49 to 12.95% (Figure 7). The highest level of m^5^C (R = 12.95%) was observed in seedlings that were grown from seeds with 8.8% mc (stored, control seed). Seedlings that were derived from seeds desiccated to 5.3% mc were characterized by a significantly lower level of global DNA methylation (10.40%). A stronger reduction in the m^5^C content of genomic DNA (down-methylation) to R = 6.49% was observed in seedlings that were grown from severely desiccated seeds (2.8% mc), (Figure 7).

## Discussion

### Changes in DNA Methylation during the Desiccation of Orthodox Seeds

Cytosine methylation in DNA plays a key role in the regulation of gene expression, and therefore affects cellular protein profiles [30], [31], [32]. Such changes control the growth and development of plants [11], [33], [34], [35], because DNA methylation is not only maintained through cell division but can also be modified. The level of 5-methylcytosine ranges from 6 to 30% in plants [36] and is actively carried out by specific DNA methyltransferases [37].

The mechanism by which stresses affect the epigenome is not well understood. Increases in global DNA methylation are known to inhibit gene expression and a reduction of methylation enhances gene expression [38]. Epigenetic alterations have been identified to allow plants to adapt to new conditions without affecting the DNA sequence [39]. There are many examples of genes whose expression is regulated by methylation status. Also, genes that are responsible for the reaction to stress signals have been shown to be regulated by epigenetic mechanisms [14]. So far, it is known that DNA methylation is substantially and rapidly reduced in the coding region of a glycerophosphodiesterase-like gene one hour after oxidative stress in tobacco. The reduced DNA methylation in the coding region correlates with stress induction of the glycerophosphodiesterase-like gene [15]. In addition, drought stress can reduce the level of DNA methylation in rice and this induced epigenetic change in its genome is considered as a very important regulatory mechanism which enables rice to adapt to drought [40].

At the present time, it is not well understood if changes in global DNA methylation in seeds play an important role in the regulation of genome-wide gene expression in response to environmental stress, especially desiccation. The ability of tissues to survive desiccation is commonly found in orthodox seeds. It is thought that metabolism shuts down as plant tissues dehydrate and enter into a state of suspended animation [41]. Contrary to this statement, our results showed that genome-wide changes of DNA methylation were induced in seeds after periods of severe desiccation. This study is the first report which shows that seeds can react at an epigenetic level to stress signals. Such changes may be a result of physiological-induced processes and unspecific DNA damages that are not the effects of programmed events. Unfortunately, at the present time, we cannot link this epigenetic reaction with any specific physiological response. However, it seems to be feasible that observed alterations in DNA methylation of dried seeds induce transcriptional changes during or before seed imbibition [42]. Nevertheless, we showed that the desiccation of common pear seeds from 8.2–8.8% to 4.8–5.3% resulted in a slight decline of global DNA methylation levels. However, no changes were observed for seed germination or the emergence of seedlings.

Secondly, our unique results clearly showed that the level of global DNA methylation increased significantly in highly desiccated seeds (desiccation above silica gel to 2.2–2.8% mc) compared with that of seeds desiccated to approximately 8% mc. It is known that an increase in the DNA methylation level represses gene expression [37]. Thus, it seems reasonable to consider that seeds may attempt to survive extremely unfavorable environmental conditions by using hypermethylation of DNA to reduce all vital cellular functions to a minimum.

In the present study, we revealed an important connection between global DNA methylation changes and the adjustment of orthodox seeds to severe desiccation conditions. Other authors have shown that histone methylation and deacetylation are involved in the induction of gene expression that inhibits seed germination under unfavorable environmental conditions [43], [44], [45]. In our study, we further showed that orthodox pear seeds use different epigenetic strategies when desiccated to different levels, with little demethylation occurring in response to moderate desiccation (Figure 4C), and hypermethylation occurring in response to extreme desiccation (Figure 4). Wang and co-workers [40] reported that drought stress tends to reduce the overall level of DNA methylation in the leaves and roots of rice plants. Other reports also indicate that environmental factors tend to cause demethylation of genomic DNA [15]. An opposite trend in the global level of m^5^C was observed for severely desiccated pear seeds in the present study.

Storage at 3°C of pear seeds from both provenances caused an increase in their global DNA methylation. Stored and non-stored seeds responded in a similar manner after severe desiccation to 2–3% mc (hypermethylation). This finding confirms our earlier assumption that this strategy for the modification of DNA methylation is characteristic of orthodox seeds and does not depend on the initial level of DNA methylation of the stored or non-stored seeds.

Analyses of time-dependent changes in seed viability and global m^5^C levels confirmed that a period of 1-year storage had no effect on the germination or emergence of seedlings compared to fresh, non-stored seeds. However, a global increase in DNA methylation from 3.08 to 5.49% was observed in response to storage at 3°C (Figure 5).

### Effect of Seed Desiccation on DNA Methylation of Seedlings derived from Desiccated Seeds

We showed for the first time that the level of DNA methylation is generally lower in seeds than in seedlings. Previously, it was shown that the endosperm of *Arabidopsis* seeds had lower levels of DNA methylation than the embryo [46], [47]. Moreover, DNA methylation was higher in mature leaves (needles) of *Pinus radiata* than in seedlings [48]. We demonstrated that changes in the global level of m^5^C in seeds are detectable in 3-month-old seedlings. These alterations that were observed in seedlings were reversed in comparison to those observed in seeds. A desiccation stress signal which had affected seeds reveal in seedlings as significant decline in amount of m^5^C. Seedlings derived from seeds that were dried to 2.8 and 5.3% mc contained a lower level of DNA methylation than those obtained from seeds with 8.8% mc. Moreover, severe desiccation of seeds resulted in a subsequent reduction of height in germinated seedlings.

It was previously observed that DNA methylation was lowered in two heterochromatic loci in tobacco cell suspensions that were treated with osmotic and salinity stress and this trend was reversed after cessation of the stress [49]. However, our results indicate that a desiccation stress signal affecting seeds alters DNA methylation, both in seeds and seedlings derived from these seeds, even they were not subjected to desiccation stress. The global m^5^C levels in pear seedlings that are older than 3 months still remain to be determined.

### Conclusions

We investigated the influence of desiccation on *Pyrus communis* seeds and seedlings. Our comparative studies confirmed that severe desiccation did not negatively impact seed germination rates, slightly reduced seedling emergence and resulted in a minor reduction of seedling height. However, we observed that severe desiccation of seeds caused an opposite effect in the DNA methylation of these seeds (slight increase) and in 3-month old seedlings (decrease). We concluded that orthodox seeds are capable of reacting to severe desiccation by means of epigenetic phenomena.
